# Pregnancy outcome following fetal reduction from dichorionic twins to singleton gestation

**DOI:** 10.1186/s12884-020-03076-7

**Published:** 2020-07-03

**Authors:** Gal Greenberg, Ron Bardin, Shir Danieli-Gruber, Kinneret Tenenbaum-Gavish, Anat Shmueli, Eyal Krispin, Galia Oron, Arnon Wiznitzer, Eran Hadar

**Affiliations:** 1 Helen Schneider’s Hospital for Women, Rabin Medical Center, 39 Jabotinsky Street, 4941492 Petach-Tikva, Israel; 2 Sackler Faculty of Medicine, Tel Aviv University, Tel Aviv, Israel

**Keywords:** Twins, Singleton, Reduction, Pregnancy outcome

## Abstract

**Background:**

There are still some controversies regarding the risks and benefits of fetal reduction from twins to singletons. We aimed to evaluate if fetal reduction from twins to singleton improves pregnancy outcome.

**Methods:**

Retrospective analysis of all dichorionic-diamniotic twin pregnancies, who underwent fetal reduction. Pregnancy outcome was compared to ongoing, non-reduced, dichorionic-diamniotic gestations. Primary outcome was preterm birth prior to 37 gestational weeks. Secondary outcomes included: preterm birth prior to 34 gestational weeks, gestational age at delivery, birthweight, small for gestational age, hypertensive disorders, gestational diabetes and stillbirth.

**Results:**

Ninety-eight reduced pregnancies were compared with 222 ongoing twins. Preterm birth < 37 gestational weeks (39.6% vs. 57.6%, *p* < 0.001) was significantly lower in the reduced group compared to the ongoing twins’ group. A multivariate analysis, controlling for parity and mode of conception, demonstrated that fetal reduction independently and significantly reduced the risk for prematurity (aOR 0.495, 95% CI -0.299-0.819). Subgroup analysis, similarly adjusted demonstrated lower rates of preterm delivery in those undergoing elective reduction (aOR = 0.206, 95% CI 0.065–0.651), reduction due to fetal anomalies (aOR = 0.522, 95% CI 0.295–0.926) and 1st trimester reduction (aOR = 0.297, 95% Cl 0.131–0.674) all compared to ongoing twins.

A Kaplan-Meier survival curve showed a significant proportion of non-delivered women at each gestational week in the reduced group compared to non-reduced twins, after 29 gestational weeks.

**Conclusions:**

Fetal reduction from twins to singleton reduces the risk of preterm birth < 37 gestational weeks, but not for more severe maternal and perinatal complications.

## Background

During the last 3 decades the incidence of twin gestations has more than doubled, mainly due to the use of assisted reproduction and advanced maternal age [1]. Multiple gestations carry a higher rate of adverse outcome compared to singletons [2]. There is an approximate 5-fold increased risk of stillbirth and a 7-fold of neonatal death, mostly as a sequelae of prematurity complications [3]. Twins born prior to 32 gestational weeks are at twice the risk of a high-grade intraventricular hemorrhage and periventricular leukomalacia compared to singletons born at the same gestational age [4].

Reduction of high order multiple pregnancies to diminish adverse pregnancy outcome is often recommended and a relatively common practice [5]. Limited and contradictory evidence is available on the impact on pregnancy outcome, when reduction of twins to singleton is performed. Current evidence suggest prolongation of pregnancy [6], reduced risk for preterm birth [7, 8] and higher neonatal birthweight [6, 8]; among women who underwent fetal reduction from twins to singleton, compared to non-reduced twins. In contrast, one study showed higher rates of pregnancy loss and extreme preterm delivery among women who underwent such fetal reduction [6]. Therefore, fetal reduction from twins to singleton remains controversial for its obstetrical and neonatal benefits.

The objective of our study was to evaluate whether fetal reduction of twin pregnancies to singleton is associated with a more favorable outcome for the remaining fetus.

## Methods

We conducted a retrospective cohort study in a single tertiary medical center in Israel, between 2005 and 2018. Reduced twins-to-singletons were compared to a control group of ongoing twins.

### Study population

We included all women, 18 and older, with dichorionic-diamniotic twins who underwent fetal reduction to a singleton fetus for any indication at all gestational weeks. The control group was constructed with women carrying an ongoing dichorionic twin gestation, at the end of the first trimester. This group consisted of all women in the same time frame as the study group, who underwent nuchal translucency between 11 + 0 to 13 + 6 gestational weeks, at our medical center, confirming a viable twin gestation, who did not undergo a fetal reduction procedure.

Women were excluded if the data on gestational age at delivery was unavailable.

### Data collection

Data on all women in the study and control groups, were collected from the computerized electronic health records and hospital’s medical charts.

Baseline demographic and clinical characteristics included maternal age, weight, height, Mullerian anomalies, maternal comorbidities (such as thrombophilia, diabetes mellitus, hypothyroidism, and hypertension), obstetrical history, mode of conception, gestational age at time of reduction and the indication for the reduction. Data on pregnancy outcome included gestational age at delivery, fetal sex, birthweight, mode of delivery, as well as pregnancy complications - hypertensive disorders, gestational diabetes mellitus (GDM), procedure related fetal loss and fetal death.

We evaluated women according to their risk for preterm birth, in both subgroups. High risk for preterm birth was defined according to known risk factors at time of inclusion, which included: prior preterm birth, previous cervical conization, Mullerian anomalies and cerclage.

Preterm birth was considered as any delivery occurring prior to 37 completed gestational weeks, and was categorized to either spontaneous or iatrogenic preterm birth.

Birthweight percentile was calculated according to nationally accepted growth curves, adjusted for number of fetuses, fetal sex and gestational age at delivery [9]. Small for gestational age (SGA) was defined as birthweight <10th percentile, and severe SGA defined below the 5th percentile of the appropriate growth curve.

Procedure related fetal loss was defined as fetal death diagnosed within a week from the reduction procedure.

Intrauterine fetal death (IUFD, stillbirth at or beyond 20 gestational weeks) [10], Late abortion (fetal loss at 10–20 gestational weeks) [10], GDM [11] and hypertensive disorders in pregnancy [12] were defined according to accepted guidelines.

### Fetal reduction

The procedure for fetal reduction was performed transabdominally by intracardiac injection of potassium chloride using a 20-Gauge needle until asystole was obtained. All procedures were performed by skilled physicians under real-time ultrasound guidance.

Indications for fetal reduction were categorized accordingly: 1) Due to woman’s request, without maternal or fetal indications (Elective subgroup); 2) Due to risk factors for preterm birth such as a history of preterm birth, Mullerian anomalies or prior conization (Risk factors subgroup); 3) Due to fetal anatomical malformations or chromosomal aberrations (anomalies subgroup).

Fetal reductions were also divided according to timing of the procedure, either: first trimester (< 14 weeks’ gestation), and second or third trimester (> 14 weeks’ gestation).

### Outcome measures

The primary outcome was preterm birth prior to 37 + 0 gestational weeks. Secondary outcomes, defined according to the CROWN intiative [13], included: preterm birth prior to 34 + 0 gestational weeks, gestational age at delivery, birthweight and birthweight percentile below 10th (SGA) or 5th (Severe SGA) percentile, preterm premature rupture of membranes (PPROM), hypertensive disorders, GDM and IUFD. Other neonatal outcomes, related to preterm birth per CROWN intiative - offspring neurodevelopment, gastrointestinal and respiratory morbidity, maternal and offspring infection and offspring mortality - were beyond our aim and scope and were not collected or assessed.

We also defined a composite outcome including any one of the following: preterm birth prior to 37 gestational weeks, hypertensive disorders or SGA.

### Statistical analysis

Statistical analysis was performed using SAS software (SAS Cooperation, Version 34.0, North Carolina, United States). Continuous variables were presented as mean and standard deviation, whereas categorical variables as count and percentages. We compared categorical and continuous variables between the two groups, as well as an analysis of variance for the indication and the timing of reduction. A Fisher exact or Chi-squared tests were used to compare categorical outcomes and Student’s t test or Wilcoxson for continuous variables, as appropriate. Regressions models for parity and conception were performed to control for differences in baseline characteristics between the 2 study groups and for subgroups according to timing and indication for reduction (linear regression for continuous outcomes - such as gestational age at delivery and logistic regression for binary outcomes - such as preterm birth). Incidences and odds ratios were reported with significance accepted at *p*-value < 0.05. We also constructed a Kaplan-Meier survival curve to analyze proportions of non-delivered women for each group.

## Results

The study group included 105 women with dichorionic-diamniotic twin pregnancy who underwent fetal reduction to a singleton pregnancy. Seven of these women were excluded due to missing data on gestational age at the time of delivery, leaving 98 women available for final analysis. Of them, 40 (40.8%) had the procedure done in the 1st trimester and 58 (59.2%) in the 2nd or 3rd trimester.

Mean gestational age at reduction was overall 18 + 4 weeks (Median 17 weeks, Range 11–34 weeks). Mean gestational age at reduction in the 1st trimester was 12 + 6 weeks, ranging from 11 + 0–13 + 6, and in the 2nd or 3rd trimester 21 + 6 weeks, ranging from 14 + 0–34 + 1 weeks. There were 2 late 3rd trimester reductions at 34 gestational weeks.

Sixty-six (67.35%) women underwent reduction due to fetal anomalies (39 due to major anatomical malformations and 27 due to chromosomal aneuploidies and other genetic syndromes), 10 (10.2%) due to risk factors for preterm delivery and 20 (20.4%) due to patient’s request. For 2 women the reason for reduction was unavailable. The control group of ongoing dichorionic diamniotic twins consisted of 222 women. Baseline characteristics for both groups and data regarding the procedure are shown in Table 1.

**Table 1:** Baseline characteristics, stratified for study and control groups

Baseline characteristics	Fetal Reduction ***n*** = 98	Ongoing Twins ***n*** = 222	***P***-value
Maternal Age, years	34.1 ± 5.0	33.6 ± 5.0	0.39
Body mass index^a^, kg/m^2^	24.4 ± 4.6	24.9 ± 5.7	0.6
Nulliparity	25 (25.5%)	100 (45%)	0.001
High Risk for Preterm Birth	16 (16.8%)	23 (10.4%)	0.134
**Mode of Conception:**			
Spontaneous	29 (29.6%)	88 (39.6%)	< 0.001
Controlled Ovarian Hyperstimulation	22 (22.45%)	32 (14.4%)	
In Vitro Fertilization	45 (45.9%)	102 (46%)	
**Comorbidities:**			
Mullerian Anomalies	2 (2.0%)	3 (1.3%)	0.6
Thrombophilia	6 (6.1%)	1 (0.4%)	0.004
Diabetes Mellitus	4 (4%)	7 (3.1%)	0.7
Hypothyroidism	11 (11.2%)	9 (4%)	0.02
Chronic Hypertension	0	5 (2.2%)	0.328
**Procedure Trimester:**			
First Trimester	36 (36.7%)	–	–
Second and Third Trimester	62 (63.3%)	–	–
**Indication Category:**			
Elective	20 (20.4%)	0	–
Risk Factors	10 (10.2%)	0	–
Anomalies	66 (67.35%)	0	–
Unknown	2 (2.0%)	–	–

Data presented as mean ± standard deviation for continuous variables and n(%) for categorical variables

^a^ Data available for 34 patients in the reduced group and for 123 patients in the non-reduced group

Pregnancy outcomes of the study group, compared to the control group, are presented in Table 2. Preterm birth before 37 gestational weeks was significantly lower in the reduced group vs. the non-reduced group (39.6% vs. 57.6%, *p* < 0.001). Among the preterm births in the study group and the control group there were significantly more spontaneous births than iatrogenic - 86.8% vs. 13.2, and 59.8% vs. 40.1%, respectively. Mean gestational age at delivery (36.8 ± 3 vs. 35.6 ± 2.4 weeks, *p* = 0.001), mean neonatal birthweight (2705.3 ± 708.7 vs. 2276.7 ± 543.9 g, *p* < 0.0001), cesarean delivery rate (42.9% vs. 77.5%, *p* < 0.001) and the composite outcome (39.8% vs. 63.5%, *P* < 0.001) were all significantly favorable in the reduced vs. the non-reduced group, respectively.

**Table 2:** Pregnancy outcome, stratified for study and control groups

Outcome	Fetal Reduction ***n*** = 98	Ongoing Twins ***n*** = 222^**a**^	***P***-value
Gestational Age at Delivery, weeks	36.8 ± 3	35.6 ± 2.4	< 0.001
**Preterm birth < 37 weeks:**	38 (39.6%)	128 (57.2%)	< 0.001
Spontaneous^b^	33 (86.6%)	76 (59.4%)	< 0.001
Iatrogenic^b^	5 (13.6%)	52 (40.6%)	< 0.001
**Preterm birth< 34 weeks:**	12 (12.5%)	41 (18.5%)	0.2
Spontaneous^b^	11 (91.7%)	27 (65.8%)	0.06
Iatrogenic^b^	1 (8.3%)	14 (34.2%)	0.06
Preterm Premature Rupture of Membranes	11 (11.6%)	35 (15.8%)	0.09
Neonatal birthweight^a^, grams	2703.6 ± 708.9	2193.1 ± 559.9	< 0.001
Neonatal birthweight^a^, centiles	44.9 ± 27.5	44.2 ± 25.9	0.8375
Birth weight < 10th percentile^a^	9 (9.2%)	41 (9.2%)	0.08
Birth weight < 5th percentile^a^	6 (5.1%)	20 (4.5%)	0.6
Gestational Diabetes Mellitus	9 (9.2%)	25 (11.3%)	0.55
Hypertensive disorders in pregnancy	2 (2.0%)	12 (5.4%)	0.24
Composite outcome	39 (39.8%)	141 (63.5%)	< 0.001
**Mode of Delivery:**			
Vaginal Delivery	49 (50.0%)	37 (16.7%)	< 0.001
Assisted Delivery	5 (5.1%)	10 (4.5%)	
Cesarean Delivery	42 (42.9%)	175 (79.0%)	
**Labor onset:**			
Spontaneous onset	52 (53.1%)	55 (24.8%)	< 0.001
Induction of labor	8 (8.2%)	21 (9.5%)	
No trial of labor	36 (36.7%)	145 (65.3%)	
5-min Apgar < 7	2 (2.0%)	1 (0.22%)	0.592
Late Abortion	0	1 (0.45%)	0.551
Intra Uterine Fetal Death	0 (0)	2 (0.9%)	0.556
Procedure Related Loss	2 (2.0%)	0 (0)	–

Data presented as number (%) or as mean ± standard deviation

^a^ For the non-reduced group, neonatal outcomes are presented for 444 newborns

^b^ Presented as percentage out of the preterm births

The rates of preterm birth < 34 gestational, PPROM, SGA or severe SGA, GDM and hypertensive disorders were not significantly different between the reduced and the non-reduced groups. The rate of neonatal birthweight <10th percentile was not significantly different in any of the subgroups.

There were 2 (2.0%) events of procedure related loss, 4 and 5 days following the procedure. In the reduction group there were no late abortions or IUFDs, while in the control, there were 2 (0.9%) events of IUFD, after 20 weeks gestation, of one of the fetuses and 1 (0.45%) event of late abortion at 17 weeks gestation of one of the fetuses.

There were 16 (16.8%) and 23 (10.4%) women who were considered at high risk for preterm birth, among the study and the control groups, respectively. The rate of preterm birth prior to 37 gestational weeks (81% vs. 56%, respectively, *p* = 0.078), prior to 34 gestational weeks (18.8% vs. 34.8%, *p* = 0.27) and gestational age at delivery (34.78 ± 3.6 vs. 34.70 ± 3.5 weeks, *p* = 0.945) were similar between these two subgroups (Table 3).

**Table 3:** High risk for preterm birth subgroup, stratified for study and control groups

	Fetal Reduction ***n*** = 16	Ongoing Twins ***n*** = 23	***P***-value
Gestational Age at Delivery, weeks	34.78 ± 3.6	34.70 ± 3.5	0.945
Preterm birth < 37 weeks	13 (81.2%)	13 (56.5%)	0.07
Preterm birth < 34 weeks	3 (18.8%)	8 (34.8%)	0.27

A multivariate analysis, controlling for parity and mode of conception, demonstrated that fetal reduction independently and significantly reduced the risk for preterm birth prior to 37 gestational weeks (aOR 0.495, 95% CI 0.299–0.819).

We also performed a subgroup analysis for our primary outcome (preterm birth < 37 weeks) and gestational age at birth, according to indication and timing of the procedure (Table 4). Compared to ongoing twins (we demonstrate a lower rate of preterm birth for 1st trimester reductions (30.0% vs. 64.4%, *p* = 0.003), elective reductions (25.0% vs. 64.4%, *p* = 0.007) and reductions due to anomalies (40.6% vs. 64.4%, *p* = 0.026). We noticed a 2-week and 1-week prolongation in the mean gestational age at delivery between the following subgroups - 1st trimester reduction (37.3 weeks) and 2nd or 3rd trimester reductions (36.4 weeks) compared to ongoing twins control group (35.3 weeks), respectively.

**Table 4:** Primary outcome (delivery < 37 weeks) and gestational age at delivery, sub-grouped by timing and indication of procedure

	Timing of Fetal Reduction		Indication for Fetal Reduction			Non-reduced Twins (***n*** = 222)	***P*** values				
	1st Trimester (***n*** = 40)	2nd/3rd Trimester (***n*** = 58)	Elective (***n*** = 20)	Risk Factors (***n*** = 10)	Anomalies (***n*** = 66)						
							(a)	(b)	(c)	(d)	(e)
Gestational age at procedure, weeks	12.7 ± 0.62	20.3 ± 5.4	13.0 ± 0.6	13.1 ± 1.6	21.3 ± 6.0	–	–	–	–	–	–
Gestational age at delivery, weeks	37.3 ± 3.1	36.4 ± 2.9	37.7 ± 2.2	35 ± 4.5	36.7 ± 2.9	35.3 ± 2.6	<.0001	0.006	0.0002	0.797	0.0003
Delivery < 37 weeks	12 (30.0%)	26 (46.4%)	5 (25.0%)	7 (70.0%)	26 (40.6%)	143 (64.4%)	0.003	0.124	0.007	0.402	0.026

Data presented as mean ± standard deviation or number (%)

*p*-value (a) - compares reduction at 1st trimester to non-reduced twins, adjusted to parity and conception

*p*-value (b) - compares reduction at 2nd/3rd trimester to non-reduced twins, adjusted to parity and conception

*p*-value (c) - compares reduction due to elective reasons to non-reduced twins, adjusted to parity and conception

*p*-value (d) - compares reduction due to risk factors for preterm delivery to non-reduced twins, adjusted to parity and conception

*p*-value (e) - compares reduction due to anomalies to non-reduced twins, adjusted to parity and conception

The subgroup analysis, by a logistic model, adjusted for parity and conception also demonstrated significant differences in favor of those who had an elective reduction (aOR = 0.206, 95% CI 0.065–0.651) or a reduction due to fetal anomalies (aOR = 0.522, 95% CI 0.295–0.926) compared to women with ongoing twins, and not to reductions due to risk factors for preterm birth (aOR = 1.830, 95% CI 0.444–7.537). Also, a lower risk for preterm birth was noted among women who had a 1st trimester reduction (aOR = 0.297, 95% Cl 0.131–0.674) but not for 2nd or 3rd trimester reductions (aOR = 0.636, 95% Cl 0.357–1.133) compared with ongoing twins (Table 5).

**Table 5:** Adjusted odds ratio for the primary outcome (delivery < 37 gestational weeks), for overall and subgroups by timing and indication of reductions

Outcome and Group	Adjusted Odds Ratio	95% Confidence Interval
Delivery < 37 weeks for any fetal reduction	0.495	0.299–0.819
Delivery < 37 weeks for 1st Trimester reductions	0.297	0.131–0.674
Delivery < 37 weeks for 2nd/3rd trimester reductions	0.636	0.357–1.133
Delivery < 37 weeks for reductions due to elective reasons	0.206	0.065–0.651
Delivery < 37 weeks for reductions due to risk factors	1.830	0.444–7.537
Delivery < 37 weeks for reductions due to anomalies	0.522	0.295–0.926

**Fig. 1:**
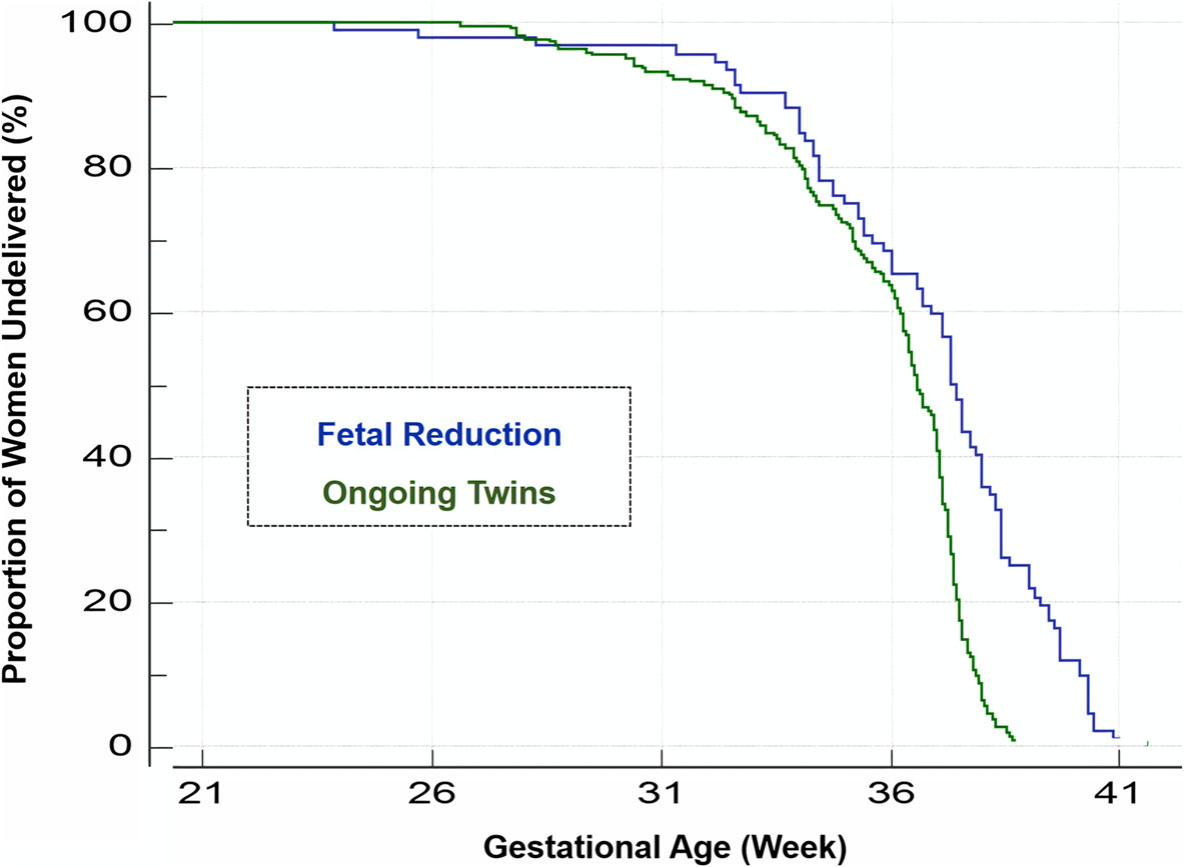
Kaplan-Meier survival curve demonstrating the relation between deliveries and gestational week for each group

A Kaplan-Meier survival curve (Fig. 1) was constructed to analyze proportions of non-delivered women for each group. Following reduction there was a significant proportion of non-delivered women at each gestational week, compared to the non-reduced twins, after 29 gestational weeks.

## Discussion

In this study we compared obstetrical outcome of twins’ gestation reduced to singleton to twins managed expectantly. Our main findings suggest that twin-to-singleton reduction is associated with decreased prematurity; fetal reduction prolongs gestation in approximately 1 week; elective reductions, reductions due to fetal anomalies and reduction at the 1st trimester are associated with a more favorable outcome than ongoing, non-reduced, twins.

The benefits and drawbacks of fetal reduction from twins to singleton have been previously studied. Gupta et al. [7] and Haas et al. [8] reported similar findings regarding the lower risk for preterm birth < 37 gestational weeks following fetal reduction. Vierra et al. [14] compared 250 twin-to-singleton elective reductions with 605 ongoing twin gestations, all preformed prior to 15 gestational weeks with supportive results to our study. Following fetal reduction superior pregnancy outcome include a lower risk of preterm birth prior to 37- or 34-weeks’ gestation and preeclampsia, without reducing the risk of growth restriction, placental abruption or GDM. Van de Mheen et al. [6] reported a 2 weeks prolongation of pregnancy - from 37.1 to 38.9 weeks, among twins who underwent reduction compared to non-reduced twins. It is interesting to note that our results indicate approximately 40% risk of preterm delivery < 37 gestational weeks in the singletons, which is in contrast to previous studies reporting a rate of 10% [7, 8] and 19% [15].

In our study, the rate of preterm birth < 34 gestational weeks was not significantly different between groups, presumably due to a small sample size. Evidence for which is shown in the Kaplan-Meier survival curve, demonstrating that following 29 weeks of gestation, women with twins have a higher chance to deliver, at each gestational age, compared to women who underwent reduction, and that up to 29 gestational weeks, the risk is similar. In contrast to our findings, Van de Mheen et al. [6] reported in their study that until 35 gestational weeks women who underwent reduction had a higher chance to deliver than women with twin pregnancy.

We included in our study women who underwent reduction for any indication at all gestational weeks. Elective reductions and reductions due to fetal anomalies, were associated with a significant lower risk for preterm birth < 37 gestational weeks, as were reductions at the 1st trimester, compared to the non-reduced group. This advantage was not detected in women who had their reductions done due to risk factors for preterm birth or had the reduction at 2nd or 3rd trimester. Our results were significant even when adjusted for parity and conception. In order to examine if fetal reduction can benefit women who are high risk for preterm birth, we compared between those women in the two groups and found that there was no significant difference between the rate of preterm birth, or gestational age at delivery, among those subgroups. This may be because of the small sample size, or since the fetal reduction does not compensate for other preexisting risk factors for preterm birth.

Also, we did not find any difference in terms of gestational age at delivery between first trimester reductions and second or third reductions, although those who had a 1st trimester reduction delivered at 37 weeks compared to 36 weeks in those with a later reduction. This is in contrast to other publications, Hasson et al. [15] found that reductions beyond 15 gestational weeks, results in higher incidence of late abortion and preterm delivery compared to reductions before 15 gestational weeks. Yaron et al. [16] reported that procedures before 14 weeks of gestations were associated with a lower rate of preterm deliveries, longer gestation and higher birthweight, compared to procedures performed after 14 weeks. However, both Gupta et al. [7] and Van de Mheen et al. [6] did not find any significant differences in pregnancy outcome between different indications or timing of reductions.

Birthweight <10th percentile was not significantly different between the reduction group and the ongoing twins’ group, nor was it different between any of the subgroups evaluated regarding indication and timing of procedure. Significant difference regarding birthweight <10th percentile in favor of the reduction group was reported only by Gupta et al. [7] We were able to show higher birthweight in the reduced group compared to the ongoing twins, this is in concordance with the findings of Haas et al. [8] and Van de Mheen et al. [6].

We report 2 events (2.2%) of procedure-related pregnancy loss, 4 and 5 days following fetal reduction, both of which underwent the reduction beyond the first trimester, at 18 and 22 gestational weeks. This loss rate is similar to what is known in the literature, in 2014 Evans et al. published a 25 years overview of fetal reductions and reported 2.5% fetal loss rate following reductions from twins to singleton [17]. There were no events of IUFD, unrelated to the procedure, in the study group. This is compared to 2 (0.9%) events of IUFD after 20 weeks gestation of one of the fetuses in the non-reduced group and 1 (0.45%) event of late fetal abortion.

It is interesting to note that in our study there was a 42.9% rate of cesarean deliveries among our study group, which is higher than the average cesarean rate among our general population. It can be explained by the fact that our study population is made out of older women with IVF pregnancies, with a liberal policy for maternal requested elective surgery as well as a high rate of repeated cesarean deliveries and malpresentations.

Fetal reductions cannot be discussed without considering ethical aspects, especially in twin-to-singleton procedures. It is debatable whether fetal reduction is ethically justified and it remains controversial whether this option should be discussed with patients, and under what, if not all, circumstances. The reason for reduction, be it medical, psychological, financial or social, should be addressed and taken into consideration when weighting it against the risks and clinical benefits in reducing twins to singleton. One can argue that a reduction from a higher order multifetal pregnancy is more appropriate as the potential gain is greater and the clinical outcome is significantly better after reduction, while reducing twins to singleton only marginally improves pregnancy outcome, in a magnitude that may not justify to reduce one of the fetuses. In 2017, the ACOG committee on ethics regarding multifetal pregnancy reduction concluded this subject with the recommendations that only the patient can weigh the relative importance of the medical, ethical, religious and socioeconomic factors and determine the best course of action for her unique situation [18]. This, as well as other studies, provide important information for appropriately counselling the patients, particularly when considering a reduction of a non-anomalous twin.

Importantly, this also reiterates the importance of avoiding iatrogenic multiple gestations and to appropriately adhere to single embryo transfer policy in order to prevent twin gestations, fetal reductions and their consequences.

### Strength and limitations

Our study, has a large cohort compared to other publications, accordingly we were able to subgroup our population according to indications and timing of reduction. Accordingly, we were able to contribute novel data where controversies and major knowledge gaps still exists. Nevertheless, our study is not without limitations mainly due to its retrospective design, rendering some parameters unknown or unavailable - especially neonatal morbidity outcomes. Also, counselling for elective fetal reduction to women in the control group, may introduce bias, as it was done according to physician’s discretion and not as routine practice. For specific indications, subgroups or outcomes our cohort may still have been underpowered to detect existing benefits.

## Conclusion

Fetal reduction from twins to a singleton pregnancy reduces the risk of preterm birth prior to 37 gestational weeks, but not for more severe maternal and perinatal complications. As this may be attributed to our sample size, larger cohorts are needed to further evaluate these associations. Be that as it may, fetal reduction from twins to singleton should at least be discussed with the expecting couple as an available management option for them to consider, with the balance between risks and benefits, with the attributed evidence, clearly and simply manifested.

## Data Availability

The datasets used and/or analyzed during the current study are available from the corresponding author on reasonable request.

## Abbreviations

SGA: Small for gestational age
GDM: Gestational diabetes mellitus
IUFD: Intra-uterine fetal death
